# Perceived Benefits and Barriers of a Community-Based Diabetes Prevention and Management Program

**DOI:** 10.3390/jcm7030058

**Published:** 2018-03-13

**Authors:** Samantha Shawley-Brzoska, Ranjita Misra

**Affiliations:** School of Public Health, West Virginia University, Morgantown, WV 26506, USA; ramisra@hsc.wvu.edu

**Keywords:** diabetes, lifestyle modifications, intervention, benefits, barriers, health literacy

## Abstract

This study examined the perceptions of benefits of and barriers to participating in a community-based diabetes program to improve program effectiveness. The Diabetes Prevention and Management (DPM) program was a twenty-two session, 1-year program, modeled after the evidence-based National Diabetes Prevention Program and AADE7 Self-Care Behaviors framework. Community-based participatory research approach was used to culturally tailor the curriculum. Participants included overweight or obese adults with dysglycemia. A benefits and barriers survey was developed to gather information on participants’ perception of the program, as well as information on demographics and health literacy levels. Eighty-nine adults participated in the DPM program (73% females; 62% diabetic; 77% had adequate health literacy); 79% of participants completed the benefits and barriers survey. Principal component analysis indicated two components representing benefits (Cronbach’s α = 0.83) and barriers (α = 0.65). The majority perceived high benefits and low barriers to program participation; benefits included helpful interaction with health coach or program leader (73%), improved lifestyle modification (65%) due to the program, and satisfaction with the program (75%). Open-ended questions confirmed themes related to benefits of program participation, suggestion for programmatic improvements as well as barriers to participation. Participant feedback could be used to guide interventions and tailor future program implementation.

## 1. Introduction

Diabetes affects 30.3 million Americans (9.4% of the population) and contributes to early morbidity and mortality [1]. In addition, 84.1 million Americans or 33.9% have prediabetes [1]. Diabetes is especially prevalent in Appalachian areas [2], particularly West Virginia, the only all-Appalachian state. West Virginia (WV) has one of the highest rates of diabetes (15.3%) and pre-diabetes (35%) among the 53 US states and territories [3,4]. This may be due in part to the majority of the residents residing in rural areas (WV is the 3rd most rural state in the country) [5] and low socioeconomic status, considering that diabetes is highest among individuals who earn a household income of less than $15,000 per year, and that 17.9% of West Virginians are below the poverty level [6]. Other factors that contribute to the high rate of diagnosed and undiagnosed diabetes and pre-diabetes in WV include geography, lack of access to quality care, the aging population, and the Appalachian culture of fatalism [7]. In addition, healthy lifestyle practices are also poor; obesity, physical inactivity, and smoking are major contributing factors to diabetes [1]. Importantly, if not properly managed, dysglycemia (individuals with diabetes and pre-diabetes) can lead to a variety of severe complications, including but not limited to heart disease, stroke, hypertension, blindness and eye problems, kidney disease, nervous system disease, and amputation [8]. In WV, diabetes-related diseases such as heart disease, hypertension and stroke are the number 1, 2 and 5 causes of death, respectively [4]. 

Dysglycemia presents a significant challenge for individuals, communities, and healthcare systems due to increased risk for disease and comorbid conditions associated with health care costs [9,10,11]. Medical expenditure was 2.3 times higher for individuals with diabetes than without and healthcare costs associated with diabetes was $245 billion in 2012 [12]. Hence, prevention and education efforts are currently focused on preventing or delaying the early onset of diabetes and its complications. Prevention is one of the most effective public health strategies for reducing the prevalence of diabetes and improving successful disease self-management. Previous evidence-based lifestyle programs, such as the Diabetes Prevention Program (DPP) [13], Da Qing Study [14], Swedish Malmo Study [15], Finnish Diabetes Prevention Study [16], and United Kingdom Prospective Diabetes Study [17], have shown that diet, physical activity, and behavior modifications can combat the rising number of individuals with prediabetes; lifestyle modification is also a critical component of diabetes self-management [18]. 

While the translation of lifestyle interventions is often recommended for reducing the risk for diabetes and its complications, adequate dissemination of diabetes prevention and management programs in WV (e.g., the DPP, WV Dining with Diabetes program, Chronic Disease Self-Management Program (CDSMP), and Diabetes Self-Management Program (DSMP), and Diabetes Self-Management Education) is less than desired [19]. Understanding the perceived benefits and barriers of participation is critical if we are to improve prevention efforts in WV communities. Rigorous and supportive research on factors that motivate or deter individuals with dysglycemia to participate in lifestyle interventions is currently lacking in this medically underserved state. Furthermore, very little diabetes research has shown adequate dissemination, the use of qualitative techniques, or the understanding of the underlying benefits and barriers involved with lifestyle programming.

Examining the role of benefits and barriers is important in understanding the effectiveness of community-based diabetes programs. Hence, the purpose of this exploratory study was to develop an instrument for assessing the benefits of a community-based diabetes prevention and management (DPM) program. These results can be used to tailor the curriculum for Appalachian culture, and can help with accessibility to diabetes programs in community settings, which may represent a novel way of improving and implementing effective programs in WV. Furthermore, results from this research can be used to tailor new and available programs for improved program adherence, utilization, and behavior changes as well as increase health provider referrals to lifestyle programs in West Virginia communities. 

The purpose of the study was to: (1) Determine the participants’ perceived barriers for engaging in the DPM program; (2) Determine the benefits of program participation; and (3) Assess the satisfaction with various components of the program (if any).

## 2. Experimental Section

### 2.1. Data Source, Eligibility and Recruitment

The original data source was the Diabetes Prevention and Management (DPM) program from 2014 through 2016. The DPM was implemented in twenty-two sessions over 12 months in two Appalachian churches, and was modeled after the evidence-based National DPP and modified to include diabetes management sessions using the American Association of Diabetes Educators Self-Care Behaviors framework (AADE7) [13,20]. The program emphasizes goal-setting and self-monitoring to make lasting improvements in nutrition and physical activity; moderate weight loss; building self-efficacy and social support for sustainable lifestyle changes; and overcoming barriers to maintaining weight-loss and lifestyle changes [21,22]. Specifically, program sessions included, but were not limited to, the following information: healthy eating, physical activity, stress management, keys to eating out, mindful eating, problem solving and medication adherence. Program participants could attend all program sessions, and those who missed a session had the opportunity to review a closed YouTube channel that had the program session recordings. The DPM program utilized a prospective study design to evaluate the delivery of an evidence-based, non-pharmacological, behavioral lifestyle intervention. Individuals who were at risk of developing diabetes (i.e., prediabetes stage) or were diagnosed with diabetes, based on survey screening questions or fasting blood glucose, were recruited for the study. Eligible individuals had to be aged 18 years or older, with a BMI of equal to or greater than 25 kg/m^2^, and had to be motivated to meet the Surgeon General’s recommended physical activity of at least 150 min per week. Potentially eligible participants were recruited from the greater Morgantown and Charleston, WV areas using convenience and snowball sampling due to the nature of the program. These two areas have several hospitals, Federally Qualified Health Centers, free clinics, churches and other community-based organizations (e.g., Young Men’s Christian Association) from which participants were successfully recruited for the program. Participants were also recruited through community advertisements such as flyers, church bulletins, email lists, and community presentations. The study protocol was approved by West Virginia University Institutional Review Board.

### 2.2. Benefits and Barriers Survey

Benefits and barriers were assessed using a 33-item survey questionnaire developed by the researchers. The survey examined participants’ perception of benefits of and barriers to participation in the DPM program. The survey questions sought information on the following: barriers to engaging in the program, benefits reported for program participation, satisfaction with various components of the program (if any), and demographic information of the participants (medical history, family history of disease, health literacy, and physical health and mental health wellbeing). In addition, individuals had the opportunity to respond, using open-ended questions, on specific benefits and barriers, provide suggested improvements for the program, and identify the most interesting components that may lead to improvement of community-based diabetes lifestyle programs.

We used a modified Centers for Disease Control clinical data form to identify the medical history of diseases, including prediabetes, diabetes and heart disease [23]. Self-rated general health status was measured by questions modified from the WV Ruby Memorial Hospital Health Questionnaire; the question asked, “Compared to other people, how do you rate your physical health?”, with response choices ranging from 1 to 5, where 1 = Poor and 5 = Excellent [24]. Self-rated mental health was measured by the question “Compared to other people, how do you rate your mental health?”, with response ranging from 1 to 5, where 1 = Poor and 5 = Excellent. In addition, health literacy was measured by three validated questions developed by Chew et al.: (1) “If you need to go to the doctor, clinic or hospital, how confident are you in filling out forms by yourself?”; (2) “How often do you have someone (family member or staff at the clinic or hospital) help you read health or medical forms?”; and (3) “How often do you have problems learning about your medical condition because of trouble understanding written health information?” [24,25]. Response options ranged from 1 to 5 for each question, where 1 = Not at all confident or Always and 5 = Extremely confident or Never. Thus, higher scores indicate adequate health literacy.

The remainder of the survey questions were developed and modified from two reliable and valid surveys which were the Community Health Awareness of Diabetes (CHAD) and the Summary of Diabetes Self-Care Activities (SDSCA) measure, along with previous lifestyle programming experience [26,27]. These specific questions identified the benefits and barriers of the diabetes program. The benefits and barriers program questions used Likert response items (1 = Not applicable, 2 = Strongly Disagree, 3 = Disagree, 4 = Neutral, 5 = Agree, and 6 = Strongly agree) and an open-ended format. The survey was reviewed by the program staff (Investigators and Health Coaches) to ensure response time, readability, and applicability. Participants were asked to complete the survey without interaction with their peers in order to reduce bias. Confidentiality was assured by using their participant ID number. The questionnaire was completed at 6 months and at the end of the program; however, the last data point was used to assess the participants’ perception. All participants were English-speaking individuals and were able to read and write in English, a norm in WV. However, study personnel assisted those participants who had questions and/or needed clarification while completing the survey.

### 2.3. Statistical Analyses

Data were analyzed using SPSS (IBM, Armonk, NY, USA) All data was double entered and compared for missingness. While 89 participants (unduplicated) were enrolled in the DPM program, the current analyses included 70 participants with complete data. Descriptive characteristics for the total sample are provided. Cronbach’s alpha of this new scale was used to assess reliability or internal consistency, and principal component analysis (PCA) with a varimax rotation was conducted to examine the factorial structure of the constructs, with eigenvalues greater than 1. An eigenvalue of 1 or greater indicates that the factor possesses at least as much total variance as is contained in a single item. PCA was computed by correlating the score for each scale item with the total score for each observation, and by comparing that to the variance for all individual item scores. Model refinement and assessment were carried out iteratively. Following the PCA, findings were quantified and assessed through descriptive analyses. Additionally, the open-ended format for some questions allowed triangulation of the quantitative and qualitative feedback on participants’ perceived program benefits and barriers.

## 3. Results

### 3.1. Demographics

Table 1 presents descriptive statistics on the total sample, which included 89 unduplicated participants. Participants were primarily female (73%) and aged 20 to 83 years (mean age = 58.51 ± 0.26 years). The majority (62%) of the participants with diabetes were of low socioeconomic status, had a college degree or higher (69.7%) and an average income (45% < $50,000). Both newly diagnosed and those with longer duration of diabetes were enrolled for the intervention program; the mean number of years with diabetes reported was 19 ± 13.5 years, and no significant difference was found between the two groups. Furthermore, two-thirds reported that they were currently married (63%) and had family members living in their household (mean = 1.24 ± 0.30).

With respect to participants who had a genetic predisposition to diabetes; 64% indicated a family history, and over half (56%) knew someone who had been diagnosed with heart disease. Likewise, hypertension (55%) and high cholesterol (54%) were major comorbidities for this study sample. However, over 50% of participants self-rated their overall physical and mental health status to be good or excellent, and more than 77% of participants had adequate health literacy.

Additionally, the rate of program completion for participants was 82%. Dropout was defined as those participants who informed the team that they were withdrawing from the study or when they were unable to be contacted after missing a scheduled assessment and/or follow-up phone calls. Attendance was taken at every educational session. Several individuals started the program, but dropped out in the first 4 weeks due to instrumental reasons such as changes in health and mobility, competing family responsibilities, transportation issues, change in job, etc. Based on attendance, taken at every educational session, the average number of sessions attended was 75% (or 14 sessions). No significant difference was noted based on diabetes and pre-diabetes status.

### 3.2. Reliability of Scale

PCA identified clustering of five constructs as follows: screening/risk, location, fasting blood work, benefits and barriers. The two factors of importance for this study were benefits and barriers; eight questions loaded for benefits and five questions loaded for barriers. The benefits and barriers components were then placed in a two-component PCA analysis to further assess reliability of the scale, which is presented in Table 2. With the two-component PCA analysis, the overall Cronbach’s alpha reliability for the benefits component was 0.827 and 0.645 for barriers. These two factors accounted for 15.3 (benefits) and 21.3 (barriers) of the variance for the total sample. Questions relating to diabetes screening/risk, fasting blood work and location were excluded from the final PCA analysis for this study.

For the main variables (benefits, barriers), the mean response was 43.6 ± 3.91 and 21.5 ± 4.62, respectively. The mean scores for these two constructs indicated high benefits and low barriers with the DPM program. DPM program aided in implementing lifestyle changes for participants and educational sessions provided a positive experience for support, discussion and learning opportunities. The benefit item-mean scores ranged from 5.03 to 5.64 on a 1–6 scale (Table 3). Overall, perceived barriers to program participation and engagement was low (range = 3.76 to 4.69) and therefore included minimal areas of improvement. Most individuals were satisfied with the program and improvement in lifestyle behaviors, and this suggests that individuals rated the individual benefits and barriers items positively. Specifically, individuals with prediabetes (mean = 5.43 ± 0.528) and diabetes (mean = 5.48 ± 0.399) scored benefits positively as well.

### 3.3. Survey Results

#### 3.3.1. Benefits and Barriers

Over 70% indicated that the interaction with their personal health coach or program leaders, as well as program materials/demonstrations, were helpful and informative. The program was successful in helping 65% of individuals to modify their health behavior and make positive lifestyle changes as a result of the program. In addition, 70% used the results of blood tests completed during the program to discuss with their healthcare provider and family/friends. The majority (74%) of participants indicated that the program helped them understand the risks associated with dysglycemia and how to prevent/manage diabetes. Overall, about 75% of respondents were satisfied with the program. Specifically, transportation was not identified as a barrier for the majority of participants. While some educational sessions were done during winter months, over half of the individuals (53%) reported they did not miss a session due to weather and work priorities, family activities and personal health issues did not deter their attendance.

#### 3.3.2. Other Constructs

As mentioned previously, the constructs that were excluded from the two-construct PCA were screening/risk, location and fasting bloodwork. Among these were some interesting conclusions that helped confirm the assumption of high benefits and low barriers. For example, over half (71%, 66%) of the participants had been screened for diabetes and had been informed of their risk for diabetes by their health provider. Approximately 69% of respondents indicated that the church location used for program implementation was convenient. Furthermore, fasting blood work done at the churches during weekend mornings was not challenging for approximately 71% of individuals; over half of participants (54%) felt comfortable giving blood. The benefits and barriers constructs correlated significantly with participant’s self-rated physical health (*r* = 0.29, *p* = 0.015 & *r* = 0.366, *p* = 0.002, respectively) indicating individuals with good health perceived greater program benefits and lower barriers to participation. Interestingly, adequate health literacy was not associated with either benefits and barriers of the program.

#### 3.3.3. Open-Ended Responses

The themes that emerged related to barriers of the program were health status, work, travel and family activities. To improve the program, the participants suggested to offer more sessions, provide more recipes, more food demonstrations, and guest speakers. Some of the benefits of the program were awareness of food intake, knowledge about disease and tools to control weight, social support and encouragement. Along with the benefits, most participants thought the guest speakers, tracking, and self-motivation presentations were the most interesting parts of the program. Specific benefits and barriers items are provided in Table 4.

## 4. Discussion

This research study examined the factors that motivated or deterred participants’ active participation, as well as suggestions for improvements. Overall, the participants had a positive impression, due to helpful and informative interactions with health coaches and program leaders and improved knowledge and skills for successful lifestyle changes as a result of the program. However, scheduling conflicts with work, travel and family activities were noted as barriers to participation. Therefore, understanding participant perceptions to barriers are critical for addressing concerns for the program.

Health behavior theories cite several factors in behavioral determination that can explain or predict a specific behavior change [28]. An individual’s perception can be positive or negative, based on the perceived benefits and barriers before a behavior takes place. For example, an individual will most likely choose a behavior that has the most benefits, especially if the benefits outweigh the barriers [29]. In particular, the health promotion model suggests that engaging in a behavior is dependent on the results of the actions whether it be benefits or barriers. Benefits and barriers can be directly or indirectly motivational to an individual when it comes to behavioral change [30]. Perhaps, directly could be by personal experiences and indirectly might be the result of a previous event. Consequently, prediction of the barriers or costs related to a behavior can in turn affect the intention to engage in a behavior or their level of performance with a behavior [31]. For behavior change, barriers can directly create an obstacle prior to engaging or indirectly through decrease in commitment. 

Our results concur with prior studies that highlight the fact that program satisfaction is based on high benefits and low barriers. Interestingly, participants self-rated their health as good or excellent despite their current chronic condition(s) and a genetic predisposition to chronic diseases (based on their family history). Additionally, participants reported adequate literacy levels and socioeconomic status than most areas of WV and were confident in reading and understanding information about their health and disease condition. The program participants were similar in demographic characteristics (age, income, geographical area) to the state, but were more educated (70% with Bachelor’s degree) and had a higher percent of females [32]. Hence, our study population was representative of the state; the majority of WV counties are rural (ranging from 4–9 in Rural-Urban Commuting Area codes (RUCA)) with our study sample including participants from counties mostly rural (3–6 in RUCA codes) [33].

The program length of one-year was not regarded as a barrier, as has been reported for the National Diabetes Prevention Program, and participants conveyed the satisfaction resulting from peer and program support as they could share their feelings, issues and struggles in a non-judgmental environment, foster peer support and encouragement, and accountability from Health Coaches [34]. The benefits of the long-term program format also allowed for incremental behavioral change and continuous feedback and support for behavior modification. Program location, transportation, weather, work priorities, and family activities did not pose a barrier and participants were enthusiastic to come for the educational sessions. Given the benefits of the program, 75% reported their overall satisfaction with participating in the DPM program. 

Despite the positive feedback received on the program, there were some limitations with this study. For example, all study variables were self-reported and could have led to measurement error and misclassification bias. Specifically, determination of benefits and barriers scores were based on a self-report measure that was developed for this study. In addition, the study is limited by the small sample size and missingness in the data.

Our findings provide evidence that the DPM program is successful in helping individuals with dysglycemia to make positive lifestyle changes and self-manage their chronic conditions. Hence, the use of Health Coaches and community-based models such as the DPM program can be successful and sustainable in WV. However, while the overall benefits were identified, suggestions for improvements such as to offer more sessions, provide recipes, food demonstrations, and bringing experts can be used to tailor future diabetes prevention and management programs.

## 5. Conclusions

Findings highlight two implications for public health. First, there is a general lack of participant perceptions of community-based diabetes programs, and this study fills a gap in diabetes intervention research in Appalachia, especially in WV. Second, participation in the DPM program improved lifestyle modification while allowing for interventions to become more tailored to the community’s needs. By identifying these benefits and barriers, the program effectiveness can be improved resulting in a better quality of life for WV communities.

## Figures and Tables

**Table 1:** Descriptive statistics of categorical and continuous, demographic variables.

Variable	Categories	*n*	Percent (%)/Mean ± SD
Sex	Female	65	73
	Male	24	27
Marital Status	Not Currently Married	27	30.3
	Currently Married	56	62.9
Education	High School or Some College	23	25.8
	College Degree or Higher	62	69.7
Income	≤$50,000	40	44.9
	≥$50,000	27	30.3
Diabetes Status	Prediabetes	34	38.2
	Diabetes	55	61.8
Age		89	58.5
Household Size		66	1.24
Benefits		70	5.45
Barriers		70	4.31

**Table 2:** Two-factor PCA reliability statistics of benefits and barriers scale.

Variable	Cronbach’s Alpha	Mean	Variance	Standard Deviation	*N* of Items
Barriers	0.645	21.5	21.3	4.62	5
Benefits	0.827	43.6	15.3	3.91	8

**Table 3:** Benefits and barriers items (benefits *n* = 69, barriers *n* = 70).

Benefit/Barrier	Item	Mean	Standard Deviation
Benefit	The health coach or program leader was encouraging or helpful	5.61	0.623
Benefit	The program materials/demonstrations were informative	5.64	0.514
Benefit	I changed my lifestyle as a result of the program	5.03	0.923
Benefit	I will discuss my program results with my health provider	5.45	0.697
Benefit	I will discuss my results with my family and/or friends	5.29	0.842
Benefit	The program helped me understand the risks associated with diabetes and/or its complications	5.49	0.797
Benefit	The program helped me understand how to prevent and/or manage diabetes	5.46	0.759
Benefit	Overall, I was satisfied with the program	5.64	0.568
Barrier	Transportation was a barrier for me	4.69	1.27
Barrier	The weather kept me from attending some program sessions	4.33	1.40
Barrier	My health status kept me attending some program sessions	4.53	1.29
Barrier	My work kept me from attending some program sessions	4.24	1.55
Barrier	Family activities kept me from attending some program sessions	3.76	1.64
Barrier	Family activities kept me from attending some program sessions	3.76	1.64

**Table 4:** Benefits and barriers themes from open-ended responses.

Benefits	Barriers
Awareness of disease	Craving at work
Encouragement/motivation	Family responsibilities
Recipes	Travel activities
Lifestyle changes	Church activities
Support/relationship building	Health
Knowledge	Length of questionnaires
Reduction in medications	One meeting time
Weight loss	
Lower blood pressure	
